# Efficacy of Transarterial Chemoembolization Combined with Tyrosine Kinase Inhibitors for Hepatocellular Carcinoma: A Systematic Review and Meta-Analysis

**DOI:** 10.3390/cancers17132110

**Published:** 2025-06-24

**Authors:** Tzu-Rong Peng, Yi-Fang Weng, Ta-Wei Wu, Chao-Chuan Wu, Chia-Lu Hsu, Ching-Sheng Hsu

**Affiliations:** 1 Department of Pharmacy, Taipei Tzu Chi Hospital, Buddhist Tzu Chi Medical Foundation, New Taipei City 23142, Taiwan; tzu.rong@tzuchi.com.tw (T.-R.P.); u108534011@gap.kmu.edu.tw (Y.-F.W.); tawei@tzuchi.com.tw (T.-W.W.); 2 School of Pharmacy, College of Pharmacy, Taipei Medical University, Taipei 11031, Taiwan; 3 Department of Surgery, Taipei Tzu Chi Hospital, Buddhist Tzu Chi Medical Foundation, New Taipei City 23142, Taiwan; ngchiukwan@yahoo.com.tw; 4 School of Medicine, Tzu Chi University, Hualien 97004, Taiwan; 5 Center for Digestive Medicine, Department of Medical Research, Taichung Tzu Chi Hospital, Buddhist Tzu Chi Medical Foundation, Taichung City 42743, Taiwan; hsuchiachialu@gmail.com; 6 School of Post-Baccalaureate Chinese Medicine, Tzu Chi University, Hualien 97004, Taiwan

**Keywords:** hepatocellular carcinoma, transarterial chemoembolization, tyrosine kinase inhibitors, overall survival, progression-free survival, meta-analysis

## Abstract

Background: Transarterial chemoembolization (TACE) is the standard treatment for patients with intermediate-stage hepatocellular carcinoma (HCC); however, its survival benefits remain unsatisfactory. In this systematic review, we aimed to compare the clinical outcomes of tyrosine kinase inhibitors (TKIs) combined with TACE and TACE alone in patients with intermediate-stage HCC. Methods: A systematic review and meta-analysis were conducted following the Preferred Reporting Items for Systematic Reviews and Meta-analysis guidelines. Randomized controlled trials (RCTs) comparing TACE plus TKIs with TACE alone in patients with HCC were retrieved from PubMed, Embase, and the Cochrane Library. The primary outcomes included overall survival (OS) and progression-free survival (PFS), reported as hazard ratios (HRs) with 95% confidence intervals (CIs). Secondary outcomes included the overall response rate (ORR) and disease control rate (DCR), which were analyzed using risk ratios (RRs). Heterogeneity was assessed using the *I*^2^ statistic. Results: Fourteen RCTs were included in this meta-analysis. Compared to TACE alone, TACE plus TKIs significantly improved PFS (HR = 0.74, 95% CI: 0.59–0.93, *p* = 0.01, *I*^2^ = 87%) and the ORR (RR = 1.29, 95% CI: 1.11–1.51, *p* = 0.001), but not OS (HR = 0.84, 95% CI: 0.69–1.03, *p* = 0.10, *I*^2^ = 65%) and the DCR (RR = 1.05, 95% CI: 0.99–1.11, *p* = 0.08). Subgroup analysis showed that TACE plus TKIs significantly increased OS in patients with hepatitis B virus (HBV) infection (HR = 0.67, 95% CI: 0.51–0.88), but not in those with hepatitis C virus (HCV) infection or those without HBV and HCV infection. Moreover, patients with HBV infection, male patients, and those with a good functional status (ECOG performance status of 0) had better PFS than others. Conclusions: Compared with TACE alone, TACE combined with TKIs can significantly improve PFS and the ORR in patients with intermediate-stage HCC. Furthermore, combination treatment can significantly improve OS in patients with HBV infection, but not in patients with HCV infection. Further research is required to optimize patient selection and treatment strategies.

## Simple Summary

Liver cancer, particularly in the intermediate stage, is often treated with a procedure called transarterial chemoembolization (TACE). Although TACE is a standard treatment, its ability to improve long-term survival remains limited. Researchers have been exploring whether adding oral cancer medications called tyrosine kinase inhibitors (TKIs) to TACE can lead to better outcomes. This study analyzed data from multiple clinical trials that compared TACE alone to TACE combined with TKIs. The results showed that the combination helped delay cancer progression and improve tumor response, especially in patients with hepatitis B-related liver cancer. However, the combined treatment did not significantly extend overall survival for all patients. These findings suggest that the combination of TACE and TKIs may be more effective for specific patient groups, such as those with hepatitis B, and highlight the need for more research to identify which patients will benefit the most from this approach.

## 1. Background

Hepatocellular carcinoma (HCC) is the most prevalent form of primary liver cancer. Globally, it accounts for a substantial cancer burden, being among the top six most frequently diagnosed cancers and a leading contributor to cancer-related mortality, ranking third in associated deaths [1]. Although patients with early HCC can receive curative treatments, such as liver transplantation, surgical resection, or ablation, more than two-thirds of patients are unable to receive curative treatments when diagnosed [2,3]. Patients with multifocal intrahepatic HCC without vascular invasion or distant metastases (intermediate stage, Barcelona clinic liver cancer stage B) [4] are recommended to be treated with transarterial chemoembolization (TACE) by international societies [5,6,7,8]; however, not all patients benefit from it, with survival after TACE ranging from 36 to 45 months to 11 months [5,9,10]. Furthermore, due to the high rate of recurrence after TACE, patients with intermediate-stage HCC often require multiple TACE sessions, which can lead to deterioration of liver function and negatively affect patient prognosis [11].

TACE can induce vascular embolization and tumor ischemic necrosis and create a hypoxic environment within the tumor, leading to upregulation of hypoxia-inducible factor-1α (HIF-1α) [12,13], promoting the production of angiogenetic growth factors, such as vascular endothelial growth factor (VEGF) and platelet-derived growth factor (PDGF), and stimulating tumor angiogenesis [12,13]. Tyrosine kinase inhibitors (TKIs) can block angiogenetic growth factors to mitigate tumor angiogenesis [13]. Several studies have tested the combination of TACE and TKIs to treat patients with intermediate-stage HCC and demonstrated its therapeutic efficacy in reducing tumor volume, decreasing vascular density, and prolonging patient survival [14]. For example, the combination of sorafenib and TACE has shown a synergistic effect and can significantly improve progression-free survival (PFS) in patients with unresectable HCC [15]. Furthermore, with the emergence of new TKIs, including lenvatinib, apatinib [16], cabozantinib, regorafenib, and donafenib [17], several studies have tested the combination of new TKIs with TACE and have shown promising results. However, the efficacy and safety of TKIs combined with TACE remain unclear. The phase III TACE-2 study by Meyer et al. found no significant differences in PFS between the combination therapy and monotherapy groups (230 vs. 235 days, *p* = 0.94) [18]. The failure of their study may have been due to their definition of disease progression; new liver lesions, a natural characteristic of HCC, may not signify true progression and should not automatically prompt treatment changes. Thus, using Response Evaluation Criteria in Solid Tumors (RECIST) 1.1 or modified RECIST may not fully capture tumor dynamics under combined therapies, such as TACE and sorafenib. Therefore, this study aimed to evaluate the effectiveness of TKI therapy combined with TACE to provide further guidance for the treatment of patients with HCC.

## 2. Materials and Methods

### 2.1. Data Sources and Search Strategy

This systematic review and meta-analysis followed the latest Preferred Reporting Items for Systematic Reviews and Meta-analysis Guidelines [19]. The study was registered in the International Prospective Register of Systematic Reviews (registration number: CRD420251000417). Two authors (Y.F.W. and T.R.P.) independently searched PubMed, the Cochrane Library, and Embase (OVID) for relevant articles published before 12 February 2025. The following terms were used for the search: (“hepatocellular carcinoma”) AND (“transarterial chemoembolization”) AND (“tyrosine kinase inhibitors” OR “sorafenib” OR “lenvatinib” OR “apatinib” OR “anlotinib” OR “orantinib” OR “brivanib” OR “sunitinib”) AND (“randomized controlled trial”).

### 2.2. Inclusion and Exclusion Criteria

The following studies were included: (1) randomized controlled trials (RCTs) published in English, (2) studies on patients diagnosed with HCC, (3) studies comparing TACE plus TKIs and TACE alone, and (4) studies documenting the occurrence of any clinical tumor outcomes mentioned in the literature, such as the overall response rate (ORR), disease control rate (DCR), and median OS and PFS. Studies were excluded if they (1) were animal experiments, case reports, reviews, letters, comments, or editorials, (2) were published in a language other than English, or (3) contained incomplete data.

### 2.3. Data Extraction

Data were independently extracted, analyzed, and recorded in standardized tables by two reviewers (T.W.W. and T.R.P.). The final decision was made after consultation with a third reviewer (C.S.H.), and a consensus was reached. Data extracted from each study included the first author, year of publication, study design, treatment regimen, sample size, and measured outcomes (ORR, DCR, OS, and PFS). The hazard ratios (HRs) of the time-to-event variables (OS and PFS) were extracted directly from the original studies or estimated indirectly using the number of events and the corresponding *p*-values for the log-rank statistics. For secondary outcomes, the ORR and DCR were analyzed using risk ratios (RRs) as summary statistics, with 95% confidence intervals (CIs) extracted directly or calculated.

Subgroup data were also extracted, where available, based on patient characteristics such as age, sex, hepatitis B virus (HBV), and hepatitis C virus (HCV) infection status and performance status (PS). PS was defined according to the Eastern Cooperative Oncology Group (ECOG) scale, where 0 indicates fully active and 1 indicates restricted in physically strenuous activity but ambulatory.

### 2.4. Quality Assessment of Included Studies

Two reviewers (T.W.W. and T.R.P.) assessed the quality of the included studies separately. The revised Risk of Bias 2.0 tool (version 2.0) [20] was used to classify the bias as low, unclear, or high (denoted green, yellow, or red, respectively) in each study.

### 2.5. Statistical Analyses

All statistical analyses were performed using RevMan software (Cochrane Review Manager Version 5.4, Oxford, UK) and Comprehensive Meta-Analysis software version 3 (Biostat, Englewood, NJ, USA). Survival results, such as OS and PFS, were reported as HRs and 95% confidence intervals (CIs). The ORR and DCR were calculated using the risk ratio (RR) and 95% CI. Calculations were performed using the DerSimonian–Laird random-effects meta-analysis [21] under the assumption of significant heterogeneity. Heterogeneity between studies was quantified using the *I*^2^ test, and *I*^2^ > 50% was considered to indicate substantial heterogeneity. A *p*-value < 0.10 was considered statistically significant. Publication bias was analyzed using Egger’s and Begg’s tests. All statistical analyses were performed according to the procedures in the *Cochrane Handbook for the Statistical Review of Interventions* (version 6.2) [20].

## 3. Results

### 3.1. Selection of Studies

**Figure 1:**
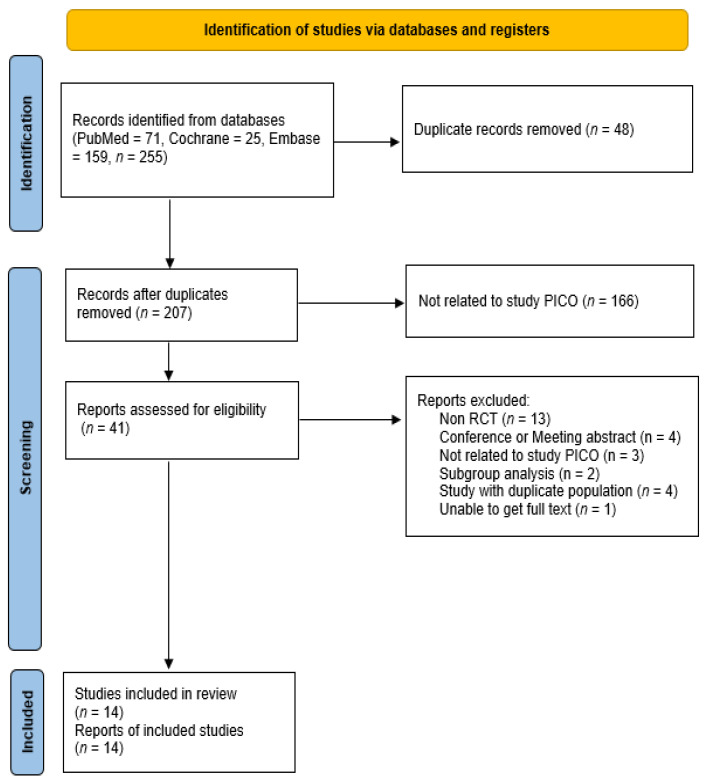
Preferred Reporting Items for Systematic Reviews and Meta-Analyses flow diagram for study selection [15]. RCT, randomized controlled trial; PICO, Patient, Intervention, Comparison, and Outcome.

We searched multiple databases (PubMed, Embase, and Cochrane databases), and 255 articles were identified for initial screening. After automatic and manual checks, 48 duplicate studies were excluded. One hundred and sixty-one articles were then screened using the abstract and title. The entire text of the 41 articles was then evaluated. Twenty-seven records were eliminated after a thorough text review because they were non-RCTs (*n* = 13), studies with duplicate populations (*n* = 4), conference or meeting abstracts (*n* = 4), not related to the study’s Patient, Intervention, Comparison, and Outcome (*n* = 3), and subgroup analyses (*n* = 2), and owing to the inability to obtain full text (*n* = 1). Finally, 14 studies were included in this meta-analysis (Table 1). The article selection flow chart is shown in Figure 1.

### 3.2. Study Characteristics and Quality Assessment

#### 3.2.1. Efficacy of TACE Plus TKIs Versus TACE Alone in Terms of Response to Treatment

**Figure 2:**
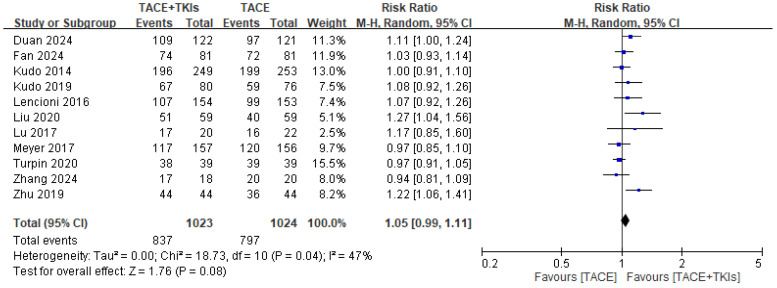
Forest plot illustrating the comparison of tumor responses to combination therapy and monotherapy, focusing on the disease control rate. TACE, transarterial chemoembolization; TKI, tyrosine kinase inhibitor; CI, confidence interval.

**Figure 3:**
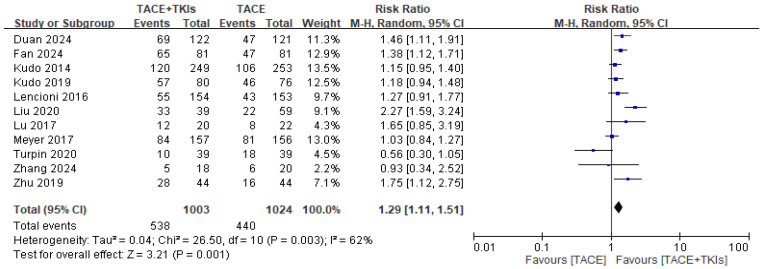
Forest plot illustrating the comparison of tumor responses to combination therapy and monotherapy, focusing on the objective response rate. TACE, transarterial chemoembolization; TKI, tyrosine kinase inhibitor; CI, confidence interval.

Two independent researchers evaluated the risk of bias among the studies, as shown in Figure S1. Eleven studies comparing TACE plus TKIs and TACE alone reported relevant data on the DCR and ORR. This meta-analysis demonstrated that, compared to TACE alone, combination therapy with TACE and TKIs increased the DCR (RR = 1.05, 95% CI: 0.99–1.11, *p* = 0.08), with low heterogeneity (*p* = 0.04, *I*^2^ = 47%; Figure 2). TACE plus TKIs significantly increased the ORR (RR = 1.29, 95% CI: 1.11–1.51, *p* = 0.001), with high heterogeneity (*p* = 0.003, *I*^2^ = 62%; Figure 3). This indicates that combination therapy with TACE and TKIs tends to result in a better response in patients with HCC compared to TACE alone.

#### 3.2.2. Efficacy of TACE Plus TKIs Versus TACE Alone in Terms of Survival Outcomes

**Figure 4:**
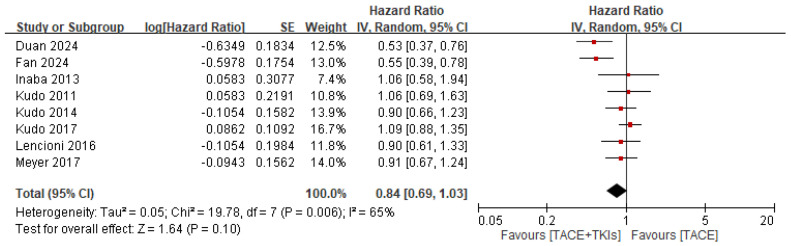
Forest plot illustrating the comparison of tumor responses to combination therapy and monotherapy, focusing on overall survival. TACE, transarterial chemoembolization; TKI, tyrosine kinase inhibitor; CI, confidence interval; SE, standard error.

**Figure 5:**
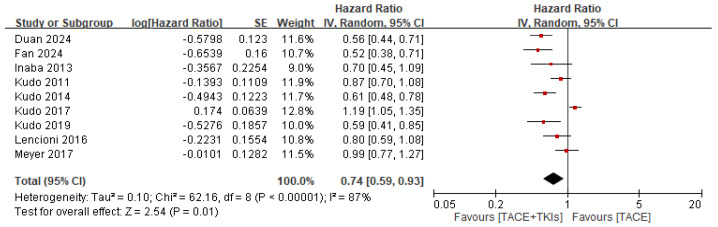
Forest plot illustrating the comparison of tumor responses to combination therapy and monotherapy, focusing on progression-free survival. TACE, transarterial chemoembolization; TKI, tyrosine kinase inhibitor; CI, confidence interval; SE, standard error.

Eight studies reported relevant data on OS. This meta-analysis showed that, in terms of OS, combination therapy with TACE and TKIs was superior to TACE alone in the treatment of HCC (HR = 0.84, 95% CI: 0.69–1.03, *p* = 0.10). However, heterogeneity was high (*p* = 0.006, *I*^2^ = 65%; Figure 4). Nine studies reported relevant data on PFS. This meta-analysis showed that, in terms of PFS, combination therapy with TACE and TKIs was significantly better than TACE alone in HCC treatment (HR = 0.74, 95% CI: 0.59–0.93, *p* = 0.01). However, the heterogeneity was high (*p* < 0.0001, *I*^2^ = 87%) (Figure 5).

#### 3.2.3. Efficacy of TACE Plus TKIs Versus TACE in Terms of Survival Outcomes: Subgroup Analysis for OS and PFS

**Figure 6:**
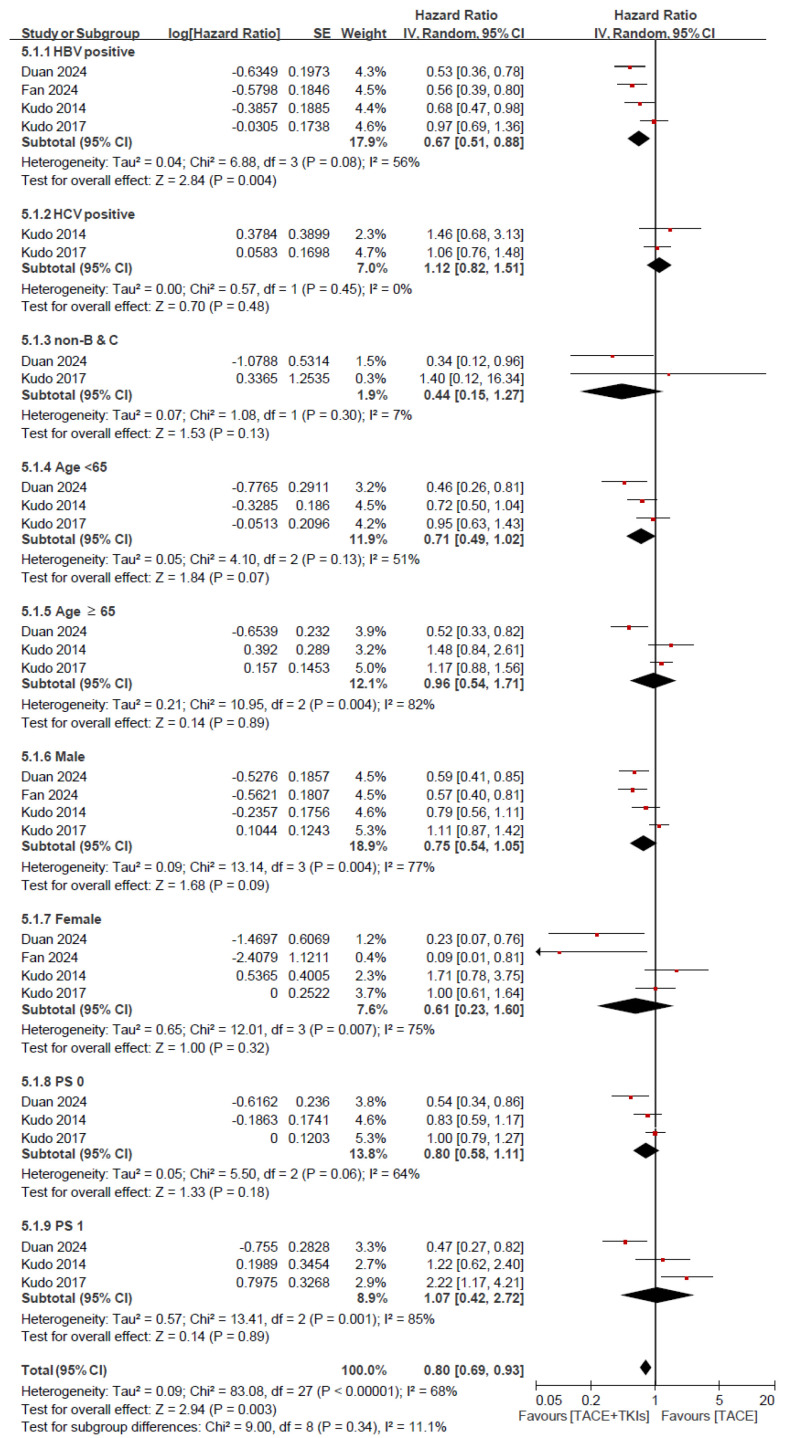
Subgroup analysis of overall survival. TACE, transarterial chemoembolization; TKI, tyrosine kinase inhibitor; CI, confidence interval; SE, standard error; HBV, hepatitis B virus; HCV, hepatitis C virus; PS, performance status; non-B & C, non-HBV-positive and non-HCV-positive.

**Figure 7:**
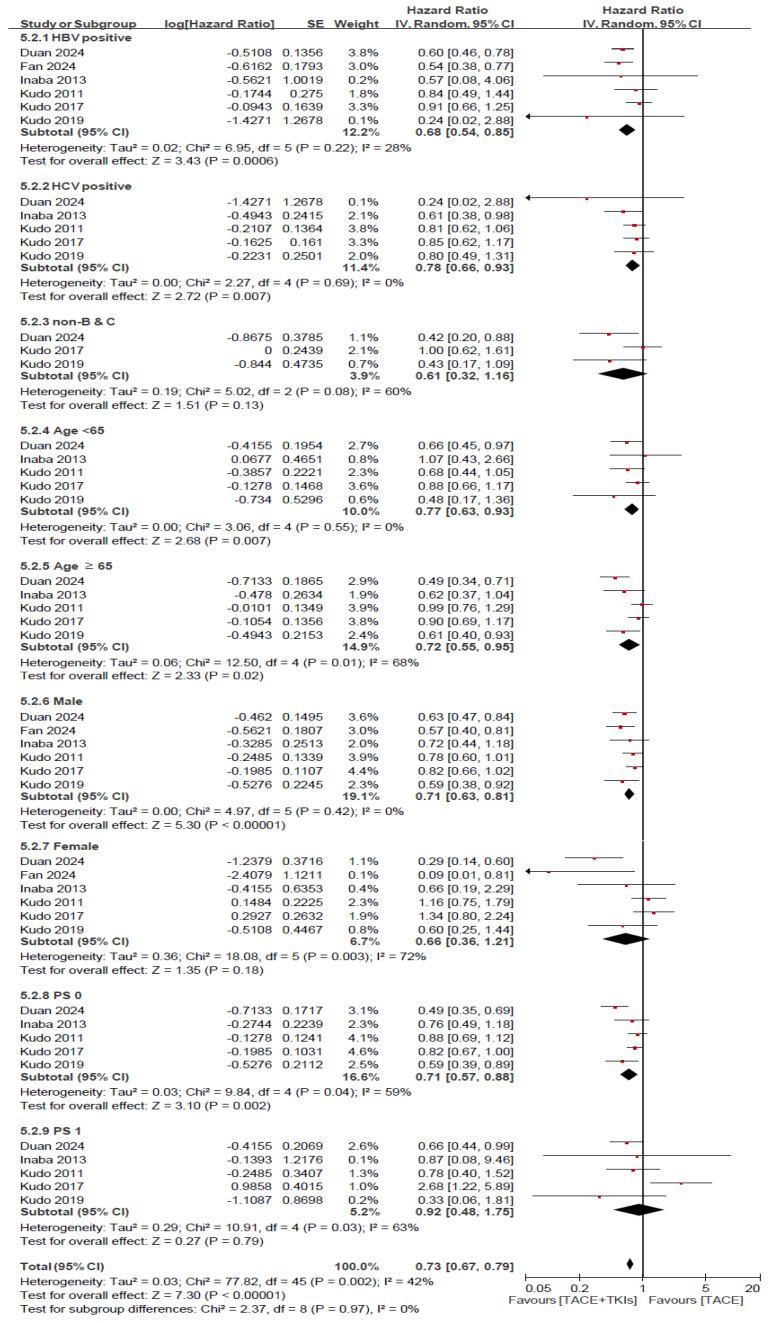
Subgroup analysis of progression-free survival. TACE, transarterial chemoembolization; TKI, tyrosine kinase inhibitor; CI, confidence interval; SE, standard error; HBV, hepatitis B virus; HCV, hepatitis C virus; PS, performance status; non-B & C, non-HBV-positive and non-HCV-positive.

We performed a subgroup analysis for OS and PFS by comparing TACE plus TKI with TACE alone according to different factors, as shown in Figure 6 and Figure 7. This meta-analysis evaluated the HRs for OS in the various subgroups according to HBV and HCV infection status (HBV-positive, HCV-positive, and non-HBV-positive and non-HCV-positive), age (<65 and ≥65 years), sex (male and female), and performance status (PS) (0 and 1). Among HBV-positive patients, a significant reduction in risk was observed (HR = 0.67, 95% CI: 0.51–0.88, *p* = 0.004). On the contrary, no significant reduction in risk was noted for HCV-positive patients (HR = 1.12, 95% CI: 0.82–1.51, *p* = 0.48). The analyses of the subgroups based on age, sex, HBV and HCV infection status, and PS did not show significant differences in the reduction of risk. Specifically, patients aged ≥65 years, both male and female participants, and those with PS of 0 and 1 did not show a significant HR (Figure 6). The meta-analysis for PFS showed a significant risk reduction in HBV-positive patients (HR = 0.68, 95% CI: 0.54–0.85, *p* = 0.0006), those aged <65 years (HR = 0.77, 95% CI: 0.63–0.93, *p* = 0.007), those aged ≥65 years (HR = 0.72, 95% CI: 0.55–0.98, *p* = 0.02), male patients (HR = 0.71, 95% CI: 0.63–0.81, *p* < 0.0001), and patients with a PS of 0 (HR = 0.71, 95% CI: 0.57–0.88, *p* = 0.002). However, no significant risk reduction in PFS was observed in HCV-positive patients, non-HBV-positive and non-HCV-positive patients, female patients, or patients with a PS of 1 (Figure 7).

#### 3.2.4. Publication Bias

**Figure 8:**
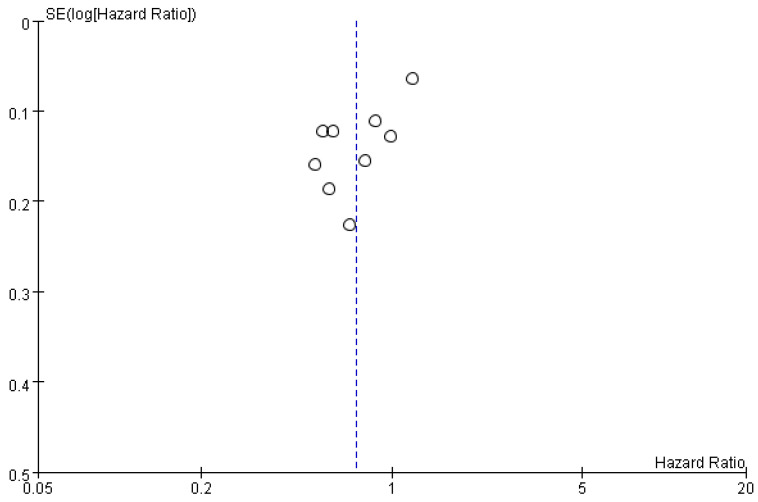
Funnel plot of potential publication bias. SE, standard error.

Visual inspection of the ORR funnel plots revealed an asymmetry (Figure 8). However, neither Egger’s nor Begg’s test provided statistical evidence of publication bias, with *p*-values of 0.441 and 0.533, respectively.

#### 3.2.5. Sensitivity Analysis

To evaluate the robustness of the meta-analysis results, a leave-one-out sensitivity analysis was performed for both ORR and PFS. For the ORR, the pooled RR ranged from 1.22 to 1.34, with the 95% confidence interval (CI) ranging from 1.07 to 1.16 for the lower bounds and from 1.39 to 1.58 for the upper bounds (Table S2). Similarly, for PFS, the pooled HR ranged from 0.69 to 0.77, with the lower and upper limits of the 95% CI ranging from 0.55 to 0.61 and 0.83 to 0.98, respectively (Table S3). These results indicate that the exclusion of any single study did not significantly affect the overall findings.

## 4. Discussion

In this systematic review, we found that combination therapy with TACE and TKIs can significantly improve PFS (HR = 0.74, 95% CI: 0.59–0.93, *p* = 0.01) and the ORR (RR = 1.29, 95% CI: 1.11–1.51, *p* = 0.001), compared to TACE alone, in patients with intermediate-stage HCC. Moreover, in the subgroup analysis, TACE plus TKIs not only improved PFS and the ORR but also significantly improved OS in male patients and those with HBV infection. These findings suggest enhanced tumor control and delayed disease progression when TKI is added to TACE, which favors its combined use in the treatment of patients with intermediate-stage HCC.

TACE can embolize tumor-feeding vessels by directly delivering chemotherapeutic agents and embolic materials to the tumor-feeding artery, inducing ischemic necrosis and creating a hypoxic microenvironment in the tumor, leading to an increase in the production of hypoxia-inducible and angiogenic growth factors [12,13]. As TKIs can block angiogenic growth factors [13], it is reasonable to add them to inhibit tumor angiogenesis and improve the therapeutic efficacy of TACE in patients with intermediate-stage HCC. Our results validated the use of this combination compared to TACE alone, and the overall analysis proved its beneficial effect on PFS and ORR, but not OS. Several factors may explain the discrepancy in the results obtained with respect to PFS and OS. First, in real-world clinical practice, patients who receive TACE alone may receive various types of treatment after progression, such as local-regional treatments, immunotherapy (e.g., programmed death (PD)-1/PD-ligand 1 inhibitors), other targeted drugs (e.g., cabozantinib and regorafenib), or additional cycles of TACE [35,36]. These subsequent treatments may potentially extend the survival of patients receiving TACE alone, although their initial treatment is less effective compared to TACE plus TKIs. Second, in RCTs, patients in both treatment groups generally received similar second-line treatments, leading to the gradual convergence of OS curves despite differences in PFS [37]. Third, although TKIs can effectively inhibit angiogenesis and tumor proliferation through the VEGF, PDGF, and fibroblast growth factor receptor pathways, tumor cells can develop adaptive resistance mechanisms through these pathways. Similarly, tumor cells may develop escape mechanisms to minimize the effects of hypoxia-induced factors (such as HIF-1α, fibroblast growth factor, or mesenchymal–epithelial transition signaling pathways) [38]. Fourth, TKIs primarily target angiogenesis rather than intrinsic tumor pathways, which means that they may delay progression but cannot effectively eliminate residual tumor cells, ultimately leading to tumor regrowth and progression after PFS. Finally, since patients with HCC may experience liver failure faster than tumor progression, PFS does not always translate into an improvement in OS in patients with HCC, particularly if liver failure, rather than tumor progression, is the main cause of death. These points explain why there was an improvement in initial tumor control (reflected in PFS), while the effects on long-term OS were limited. Several ongoing trials that test the combination of TACE with immune checkpoint inhibitors that can stimulate antitumor immune responses, rather than only targeting angiogenesis, and overcome some of the limitations mentioned above [39,40] may provide helpful information in the future.

Notably, our subgroup analysis showed that patients with HBV infection, rather than those with HCV infection, may benefit in terms of OS from combination therapy with TACE and TKIs. Because HBV-related HCC usually has higher VEGF expression, it can be more responsive to VEGF-targeted TKIs [41], resulting in a better response and greater survival benefits in patients with HBV infection who receive the combination of TACE and TKIs. Subgroup findings for HBV-positive patients are clinically relevant and warrant further discussion. HBV-related HCC has distinct molecular and pathological features compared to other etiologies, which may contribute to its differential response to targeted therapies. Specifically, HBV-related tumors have been shown to express higher levels of vascular endothelial growth factor (VEGF), a key mediator of angiogenesis, than HCV-related or non-viral HCC [41,42]. This overexpression of VEGF may make HBV-related HCC more susceptible to VEGF-targeted tyrosine kinase inhibitors (TKIs), thus improving the efficacy of combination therapy with TACE and TKIs.

Furthermore, HBV infection can activate several oncogenic pathways, such as the PI3K/AKT/mTOR and MAPK signaling cascades, which are also involved in angiogenesis and tumor proliferation [43]. Some TKIs used in the included studies, such as sorafenib and lenvatinib, target these pathways directly or indirectly, possibly contributing to a better therapeutic response in this subgroup. These molecular and histological differences underscore the importance of etiology-specific approaches in HCC management and suggest that patients with HBV-related HCC may derive greater benefit from TACE combined with anti-angiogenic agents such as TKIs. Future studies should further investigate these mechanisms and explore biomarker-driven strategies to optimize treatment selection. Furthermore, patients with a PS of 0 demonstrated a significant PFS benefit (HR = 0.71, *p* = 0.002), while patients with a PS of 1 did not. This supports the concept that patients with better baseline liver function and PS are more likely to tolerate and benefit from combination therapy [44].

Our systematic review and meta-analysis provide a focused evaluation of the efficacy of TACE combined with TKIs, specifically in patients with intermediate-stage hepatocellular carcinoma (BCLC stage B). Unlike previous reviews that included mixed populations of unresectable HCC or focused on specific embolization techniques such as DEB-TACE, our study restricts inclusion to RCTs, thus improving internal validity and minimizing confounding bias. Furthermore, we performed detailed subgroup analyses by HBV/HCV status, age, sex, and ECOG performance, uncovering a potential survival benefit, particularly in HBV-positive patients, a finding not reported in previous systematic reviews.

Our results demonstrated significant improvements in PFS and ORR with combination therapy and suggest a favorable trend in OS in HBV-related HCC. This distinction may be clinically important in HBV-endemic regions, where optimal patient selection is critical. While heterogeneity was observed in tumor response outcomes, likely due to variations in response assessment criteria (e.g., RECIST vs. mRECIST), our sensitivity analyses confirmed the robustness of the primary findings. On the contrary, previous systematic reviews included broader or more heterogeneous patient populations and study designs [45,46], which limited the specificity of their conclusions. Furthermore, they did not offer detailed subgroup analyses, particularly regarding the results of HBV-related HCC. In comparison, our findings provide more focused and clinically relevant evidence supporting the use of TACE combined with TKIs in selected patients with intermediate-stage HCC, especially those with HBV infection. More prospective studies are warranted to validate these findings, explore the underlying biological mechanisms, and optimize patient selection strategies to improve treatment outcomes.

In this study, we observed substantial heterogeneity in the analysis of tumor response to HCC treatment. We hypothesize that this heterogeneity mainly stems from differences in the criteria used in all included studies to define and evaluate tumor response. This variability not only affects the comparability of the results between studies but also poses challenges to the overall interpretation of the efficacy of the treatment. Specifically, several studies have adopted RECIST 1.1 for response evaluation [18,31,32,33,34]. On the contrary, mRECIST, which was specifically developed for HCC, places greater emphasis on the viable part of the tumor—namely, the area of arterial phase enhancement [23,24]—and is therefore more sensitive in capturing the effects of loco-regional therapies such as TACE or RFA.

For example, although Duan et al. [32] primarily used mRECIST to assess the ORR and DCR, the study also conducted sensitivity analyses using RECIST 1.1, showing comparable results between the two criteria. However, Meyer et al. [18] reported significant differences in complete response (CR) rates when comparing mRECIST and RECIST 1.1, underscoring the impact of evaluation criteria on reported results. Additionally, Kudo et al. [24] applied a modified version of mRECIST tailored for HCC (mRECIST for HCC), further enhancing the ability to detect changes in tumor viability. Other studies, such as Fan et al. [34], Lencioni et al. [25], and Lu et al. [27], also adopted mRECIST as their primary evaluation tool and reported relatively higher ORR and DCR. In summary, the diversity of tumor response evaluation criteria is a key contributor to the heterogeneity observed in this study. Future research should consider standardizing response assessment methods or, at the very least, clearly reporting the criteria used to improve cross-study comparability and clinical applicability.

In the evolving landscape of loco-regional therapies for hepatocellular carcinoma (HCC), Facciorusso et al. conducted a systematic review and meta-analysis comparing yttrium-90 radioembolization (Y90RE) and TACE [47]. Their findings demonstrated that while both modalities offer comparable overall survival and tumor response rates, Y90RE was associated with delayed tumor progression and significantly improved progression-free survival at 1 year (OR = 1.67; 95% CI: 1.10–2.55; *p* = 0.02), suggesting a potential role in improving disease control without added toxicity. These results underscore a shift toward optimizing loco-regional interventions for intermediate-stage HCC.

Based on this state-of-the-art foundation, our current meta-analysis evaluates the integration of systemic therapy, namely TKIs, with TACE. Our findings indicate that TACE combined with TKIs significantly improves progression-free survival (HR = 0.74) and objective response rate (RR = 1.29) compared to TACE alone, but does not significantly extend overall survival in the general HCC population. This pattern mirrors the observations of Facciorusso et al., [47] reinforcing the notion that improved local disease control does not always translate into prolonged survival, possibly due to subsequent therapies or the decline of liver function.

Taken together, these findings highlight the need for more personalized therapeutic approaches. Specifically, the benefits observed in our study—such as the significant survival advantage observed in patients with HBV-related HCC—suggest that etiology-specific or biomarker-driven strategies may be critical to identify patients most likely to benefit from combination regimens.

This study has several limitations. First, although 14 RCTs with more than 2000 patients were included, the statistical power to detect small but clinically significant treatment effects remains uncertain. Without a trial-sequence analysis or a predefined power calculation to assess the sufficiency of the accumulated evidence for detecting a minimally important difference, this limitation should be interpreted with caution. Second, despite the subgroup analyses, significant heterogeneity was observed in some outcomes (OS and PFS). Variability in study designs, patient populations, and treatment regimens may have affected the generalizability of the results. Different studies may have used different TKIs, dosages, or schedules, which could have influenced the overall efficacy observed. Third, most of the included studies reported short-term outcomes, such as PFS and the ORR. Long-term survival data (such as OS) were inconclusive, suggesting the need for further research with longer follow-up periods to better understand the effect of combination therapy on OS. Fourth, although Egger’s and Begg’s tests yielded negative results, it is important to recognize that these tests have limited statistical power when fewer than 15 studies are included. Therefore, the possibility of publication bias cannot be entirely ruled out and should be interpreted with caution. Finally, the included studies used different treatment regimens and doses of TACE and TKIs, which may have affected the generalizability of the findings in clinical practice. Future trials using standardized treatment protocols may provide more consistent and applicable results.

## 5. Conclusions

This systematic review and meta-analysis suggests that the combination of TACE and TKIs can improve PFS and the ORR in patients with HCC. However, its effect on OS remains inconclusive, likely due to the complex interaction between subsequent therapies, tumor adaptive resistance mechanisms, and liver reserve. Although the results indicate the potential benefit of combination therapy, the variability in treatment protocols, patient populations, and study designs limits the generalizability of the findings. Further well-designed RCTs with longer follow-up periods and standardized treatment regimens are needed to confirm these findings and better understand the long-term impact of combination therapy with TACE and TKIs in patients with HCC.

## Figures and Tables

**Table 1:** Characteristics of the studies included in the meta-analysis.

Study	Country	Intervention	Sample Size	Gender (M/F)	Age (Year)	Child-Pugh Class: A/B/C	ECOG Score: 0–1/2	BCLC Stage: A/B/C	HBV Infection	HCV Infection	Outcomes
Kudo 2011 [22]	Japan	Sorafenib + TACE	229	174/55	69	69/0/0	229/0	NA	21.1%	61.7%	OS, PFS
		TACE	229	168/61	70	70/0/0	229/0	NA	20.5%	60.7%	
Inaba 2013 [23]	Japan	Orantinib + TACE	50	39/11	≤65:39; >65:11	40/9/0 (unknown: 1)	50/0	21/24/5	2	40	OS, PFS
		TACE	51	43/8	≤65:42; >65:9	45/6/0	51/0	21/27/2	4	36	
Kudo 2014 [24]	Asia, Europe, USA	Brivanib + TACE	249	206/43	57 (21–85)	239/9/1	249/0	65/129/55	158	49	DCR, ORR, OS, PFS
		TACE	253	216/37	59 (22–85)	231/20/2	253/0	57/150/46	168	42	
Lencioni 2016 [25]	USA	Sorafenib + TACE	154	135/19	64.5	153/1/0	NA	NA	55	39	DCR, ORR, OS, PFS
		TACE	153	126/27	63.0	152/0/0 (missing: 1)	NA	NA	50	41	
Kudo 2017 [26]	Japan, South Korea, Taiwan	Orantinib + TACE	444	363/81	66.2 ± 10.2	444/0/0	444/0	158/209/74	108	193	OS, PFS
		TACE	444	364/80	65.4 ± 10.0	444/0/0	444/0	135/229/72	90	165	
Lu 2017 [27]	China	Apatinib + TACE	20	16/4	56.1 ± 10.79	18/4/0	NA	0/18/2	20	NA	DCR, ORR
		TACE	22	17/5	58.9 ± 9.38	17/3/0	NA	0/19/3	18	NA	
Meyer 2017 [18]	UK	Sorafenib + TACE	157	139/18	65 (57–71)	145/5/0 (unknown: 7)	156/NA (unknown: 1)	NA	7	15	DCR, ORR, OS, PFS
		TACE	156	138/18	68 (63–74)	148/3/0 (unknown: 5)	155/NA (unknown: 1)	NA	7	9	
Kudo 2019 [28]	Japan	Sorafenib + TACE	80	63/17	72.0 (36–85)	79/1/0	NA	27/44/9	10	38	DCR, ORR, PFS
		TACE	76	55/21	73.0 (55–86)	71/6/0	NA	33/34/9	2	53	
Zhu 2019 [29]	China	Apatinib + TACE	44	32/12	≤60:29; >60:15	38/6/0	NA	NA	36	NA	DCR, ORR
		TACE	44	34/10	≤60:25; >60:19	36/8/0	NA	NA	34	NA	
Turpin 2020 [30]	France	Sunitinib + TACE	39	36/3	66.0 (46.0–84.7)	36/2/0 (unknown: 1)	NA	NA/33/NA	1	4	DCR, ORR
		TACE	39	35/4	67.4 (43.7–84.7)	37/2/0	NA	NA/25/NA	2	4	
Liu 2020 [31]	China	Sorafenib + TACE	59	37/22	56.31 ± 9.87	43/16/0	59/0	0/30/29	NA	NA	DCR, ORR
		TACE	59	32/27	58.11 ± 10.44	48/11/0	59/0	0/36/23	NA	NA	
Duan 2024 [32]	China	Apatinib + TACE	122	100/22	57.5 ± 10.2	104/18/0	122/0	0/48/74	99	4	DCR, ORR, OS, PFS
		TACE	121	107/14	58.8 ± 11.1	101/20/0	121/0	0/40/81	106	2	
Zhang 2024 [33]	China	Anlotinib + TACE	18	16/2	62.2 ± 11.73	14/1/3	16/NA (unknown: 2)	NA/13/4 (unknown: 1)	18	0	DCR, ORR
		TACE	20	18/2	63 ± 9.44	15/3/2	18/NA (unknown: 2)	NA/12/8	18	0	
Fan 2024 [34]	China	Sorafenib + TACE	81	72/9	<50:31; ≥50: 50	81/0/0	81/0	NA	76	2	DCR, ORR, OS, PFS
		TACE	81	79/2	<50:28; ≥50: 53	81/0/0	81/0	NA	72	3	

TACE, transarterial chemoembolization; M, male; F, female; ECOG, Eastern Cooperative Oncology Group; BCLC, Barcelona clinic liver cancer; HBV, hepatitis B virus; HCV, hepatitis C virus; NA, not available; DCR, disease control rate; ORR, objective response rate; OS, overall survival; PFS, progression-free survival. Disease control rate = complete response + partial response + stable disease.

## Data Availability

All data, models, and code generated or used during the study appear in the submitted article.

## Supplementary Materials

The following supporting information can be downloaded at: https://www.mdpi.com/article/10.3390/cancers17132110/s1, Figure S1: Risk-of-bias assessments for randomized clinical trials were included in the meta-analysis; Table S1: The PRISMA checklist; Table S2: Leave-one-out sensitivity analysis for the comparison of tumor responses between combination therapy and monotherapy, with a focus on the objective response rate; Table S3: Leave-one-out sensitivity analysis for the comparison of tumor responses between combination therapy and monotherapy, with a focus on the progression-free survival.
